# The Archbishop on the National Hospital for the Paralysed and Epileptic

**Published:** 1889-06-01

**Authors:** 


### The Archbishop on the National Hospital for the Paralysed and Epileptic.

The Albany Memorial festival dinner was held on Thursday, May 23, the Lord Chancellor presiding, and His Grace the Archbishop of Canterbury being also present.

Besides being a means of affording relief to sufferers of a special class, this hospital does a great work in teaching. In 1888, 500 members of the medical profession not connected with the institution studied within its wards, and paid 1,900 visits. Of these, 123 were students, their reported attendances being 514; 28 were members of the profession in active practice; 59 came from foreign parts; 23 from Europe; and eight from many other places so far off as British Guinea, the Cape, Australia, and New Zealand.

His Grace the Archbishop of Canterbury made an eloquent appeal for funds, describing the nature of work that is done by the hospital, and assuring his hearers that a great and valuable scientific work was being done. Testimony to this was borne by letters received constantly by the institution from those who had enjoyed the advantages of its treatment, and had been brought back almost from death to life; and he knew himself how letters of this kind were confirmed by words and by the looks of those who supplied the grateful testimony. His Grace had visited the hospital recently—a mingled pain and pleasure—under the conduct of Mr. Radcliff. He really did not know whether that doctor thought him cowardly, but when asked if he would like to talk with the sufferers, he replied that the exchange of even a few words under the circumstances might be painful. But he was mistaken, and it wanted no such conversation to prove it, for it was written in the glances and the looks of these people that they felt they were being helped upwards out of despair into life again, and all bore the most affectionate and grateful testimony to the work this institution is doing. He had seen a most promising and beautiful boy struck down a life-long cripple. He had seen one that they said was "too good for the earth" suddenly stricken, and from that moment remained as one who was not of the earth, and the most touching scene he could recall was of a well-known country physican, who had been in the best of strength and spirits, going forth to do his works of mercy and kindness every day, loved by all, the helper and adviser of all, and the trusted man who received the secrets and confidences of whole families—a few weeks later he was lying on his back on his bed, weeping like a child because he should never be a man again! It was thus that helplessness and convulsions came down upon our brightest and best, and left their mysterious curse upon them. Their cases were formerly hopeless, but now, by the agency of institutions like this, such terrible visitations were ceasing to be hopeless. When his Grace was told that, in the case of a man dying from tumour in the brain, the physician can trace the exact seat of the disease, and so remove the growth by indications given by movements or non-movements of a thumb or hand, he stood amazed! Yet that appeared to be an undoubted fact. It was no stinted help they ought to bring to an institution like this, which lasted for years and years, and is worthy of the utmost devotion and attention of all people. Its resources, too, were drawn heavily upon, and it had difficulty in keeping open a sufficient number of beds to meet the demands. Again, there was a branch in which they had set on foot a system of pensions for the incurables. His Grace considered this noble, and he believed as many as seventy-five pensions were at the present time being paid. He thought they should help an institution which distributed so much relief amongst men. No less than 1,300 cities and towns were helped by the hospital, which had therefore a fair claim to the term national. On the previous Monday alone cases had been admitted from six counties, besides those from London. The annual expenditure was £12,000, and of this £8,000 was from charitable contributions. His Grace thought that they should see that this sum was provided, and even, if necessary, trebled. The hospital contained 175 beds, but three times that number were required. He hoped many who were not there in person, and whose ears were not with them, would still hear what had been said about the hospital, its work, and its needs. These simple facts would speak for themselves.

